# Solanidine‐derived CYP2D6 phenotyping elucidates phenoconversion in multimedicated geriatric patients

**DOI:** 10.1111/bcp.70004

**Published:** 2025-05-29

**Authors:** Jens Andreas Sarömba, Julian Peter Müller, Jolanta Tupiec, Anjali Roeth, Berkan Kurt, Florian Kahles, Thea Laurentius, Cornelius Bollheimer, Julia C. Stingl, Katja S. Just

**Affiliations:** ^1^ Institute of Clinical Pharmacology University Hospital RWTH Aachen Aachen North Rhine‐Westphalia Germany; ^2^ Department of General, Visceral, Pediatric and Transplantation Surgery University Hospital RWTH Aachen Aachen North Rhine‐Westphalia Germany; ^3^ Department of Internal Medicine I – Cardiology University Hospital RWTH Aachen Aachen North Rhine‐Westphalia Germany; ^4^ Department of Geriatric Medicine University Hospital RWTH Aachen Aachen North Rhine‐Westphalia Germany; ^5^ Department of Geriatrics Carl von Ossietzky University of Oldenburg Oldenburg Lower Saxony Germany

**Keywords:** biomarker, CYP2D6, pharmacogenetics, phenoconversion, phenotyping, polypharmacy, solanidine

## Abstract

**Aims:**

Phenoconversion, a genotype‐phenotype mismatch, challenges a successful implementation of personalized medicine. The aim of this study was to detect and determine phenoconversion using the solanidine metabolites 3,4‐seco‐solanidine‐3,4‐dioic acid (SSDA) and 4‐OH‐solanidine as diet‐derived cytochrome P450 2D6 (CYP2D6) biomarkers in a geriatric, multimorbid cohort with high levels of polypharmacy.

**Methods:**

Blood samples and data of geriatric, multimedicated patients were collected during physician counsel (CT: NCT05247814). Solanidine and its metabolites were determined via liquid chromatography/tandem mass spectrometry and used for CYP2D6 phenotyping. CYP2D6 genotyping was performed and activity scores (AS) were assigned. Complete medication intake was assessed. A shift of the AS predicted via genotyping as measured by phenotyping was calculated.

**Results:**

Solanidine and its metabolites were measured in 88 patients with complete documentation of drug use. Patients had a median age of 83 years (interquartile range [IQR] 77‐87) and the majority (70.5%, n = 62) were female. Patients took a median of 15 (IQR 12‐17) medications. The SSDA/solanidine metabolic ratio correlated significantly with the genotyping‐derived AS (*P* < .001) and clearly detected poor metabolizers. In the model adjusted for age, sex, Charlson Comorbidity Index and estimated glomerular filtration rate each additional CYP2D6 substrate/inhibitor significantly lowered the expected AS by 0.53 (95% confidence interval 0.85‐0.21) points in patients encoding functional CYP2D6 variants (*R* ^2^ = 0.242).

**Conclusions:**

Phenotyping of CYP2D6 activity by measurement of diet‐derived biomarkers elucidates phenoconversion in geriatric patients. These results might serve as a prerequisite for the validation and establishment of a bedside method to measure CYP2D6 activity in multimorbid patients for successful application of personalized drug prescribing.

**What is already known about this subject**

- Measurement of diet‐derived biomarkers show promising results in cytochrome P450 2D6 (CYP2D6) phenotyping.
- CYP2D6 activity is highly dependent on the CYP2D6 pharmacogene, but genotype‐phenotype mismatch may occur.
- Multimedicated geriatric patients are at an elevated risk of phenoconversion and adverse drug reactions.

**What this study adds**

- The viability of minimally invasive CYP2D6 phenotyping was demonstrated in a multimorbid geriatric cohort.
- CYP2D6 poor metabolizers could be clearly identified using this method.
- With a higher number of CYP2D6 substrates and inhibitors, the enzyme activity decreased.

## 1 INTRODUCTION

Personalized medicine aims to tailor the selection and dosing of a specific drug based on individual patient characteristics. Along with other important individual factors such as age or gender, the focus has been on the importance of genetic variability influencing drug efficacy and safety, namely pharmacogenetics.^1^ This contrasts with the traditional “one size fits all” approach where clinically important variations in human genetics are not considered.^2^

It is expected that around 25% of frequently used drugs are metabolized via **cytochrome P450 (CYP) 2D6** (CYP2D6).^3^ Many advances have been made in the field of CYP2D6‐metabolized drugs and several guidelines^4^, ^5^, ^6^, ^7^ have made recommendations on how to incorporate patient genotyping results into clinical decision making.

Adverse drug reactions (ADRs) may occur if a drug is eliminated inadequately or when active metabolites are formed at an elevated rate, especially in drugs with a narrow therapeutic range. As an example, this may happen in functional alterations of the drug‐metabolizing enzyme CYP2D6, which is genetically highly polymorphic.^8^ Clearances of drugs metabolized mainly by CYP2D6 have been shown to depend on genotypes,^9^, ^10^ alter the pharmacodynamic response to certain drugs and potentially increase ADR occurrence.^11^, ^12^, ^13^ In fact, around 90% of ADRs are estimated to be dose‐dependent^14^ and the PREPARE‐study showed that the prevalence of ADRs can be reduced by 30% if pharmacogenetic diagnostics together with dosing guidelines are implemented,^15^ although the generalizability of these findings is disputed.^16^

There are certain barriers in the implementation of pharmacogenetics, for example the lack of reimbursement by health insurance companies and uncertainties in the interpretation of diagnostic tests.^17^ This can be seen even in situations with clear available dosing guidelines based on pharmacogenetics, such as the use of antidepressants and antipsychotics.^18^

In addition, there are certain factors contributing to a potential decreased predictive power of CYP2D6 genotyping in clinical practice, such as phenoconversion, which is a genotype‐phenotype mismatch.^19^ Known causes include comorbidities and concomitant medication intake, both of which are more prevalent in older patients.^20^ This is striking because the patient group of older, multimedicated adults is frequently affected by ADRs.^21^

While some efforts have been undertaken to quantify the impact of phenoconversion on this growing vulnerable patient group,^22^ research on phenoconversion in geriatric patients taking multiple drugs has been hindered by the unfeasibility of classical probe drug approaches.^23^, ^24^

**Solanidine** and its metabolites, identified as 3,4‐seco‐solanidine‐3,4‐dioic acid (SSDA) and 4‐OH‐solanidine,^25^ have been shown to be viable biomarkers for the minimally invasive measurement of CYP2D6 activity in human body liquids^26^, ^27^, ^28^, ^29^ and to predict the CYP2D6‐mediated activation of tamoxifen.^30^ However, the viability of solanidine phenotyping in geriatric patients and the impact of phenoconversion through concomitant medication and comorbidities remain unclear.

The aim of this study was to detect and quantify phenoconversion using CYP2D6 phenotyping by diet‐derived biomarkers in a geriatric, multimedicated population.

## 2 METHODS

### 2.1 Study design

In this observational study, we investigated a cohort of 90 geriatric patients undergoing physician counsel for polypharmacy at the University Hospital RWTH Aachen, Germany. Patients were recruited during patient counsel at the interdisciplinary polypharmacy consultation service of the geriatric and the clinical pharmacology outpatient clinic (CT: NCT05247814). Patients were included if they were 70 years or older and their current drug therapy consisted of three or more drugs. They were excluded if they were classified as terminally ill by the medical staff or had less than wheelchair‐level mobility. Only first patient visits to the polypharmacy counsel were respected for analysis (baseline visits). Patients presented to the polypharmacy counsel between May 2022 and March 2024. All patients or their respective legal guardians provided written informed consent. This study was approved by the responsible ethics committee of RWTH Aachen University (393/21).

### 2.2 Clinical assessment

We fully assessed medication intake, including prescribed drugs, over‐the‐counter medications and food supplements, and documented all known comorbidities. Comorbidities were documented using the International Statistical Classification of Diseases and Related Health Problems (ICD)‐10 coding system. The Parker Mobility Score,^31^ the Timed Up and Go Test,^32^ and the Geriatric Depression Scale^33^ were used for assessments. Hand grip strength was measured with a Jamar hand dynamometer in triplicate and the best measurement was documented. Potential liver dysfunction was assessed using the Fibrosis‐4 Index score.^34^ The glomerular filtration rate (eGFR) was estimated based on creatinine measurements.^35^ In addition, the Charlson Comorbidity Index (CCI)^36^ was calculated for each patient (**Supporting Information Data** **S1**).

The medication was analysed for CYP2D6 inhibitors and substrates according to Indiana University's Drug Interactions Flockhart Table.^37^ We calculated the number of CYP2D6 substrates and inhibitors per patient. CYP2D6 substrates are known for their own inhibitory potential on CYP2D6 due to saturation effects and competitive enzyme inhibition potentially leading to “autophenocopying”.^38^, ^39^ Hence, we combined CYP2D6 substrates and inhibitors and calculated the number of uses per patient.

### 2.3 Acquisition and handling of patient samples

During patient counselling two blood samples, plasma and whole blood, were drawn into EDTA tubes and shaken gently. The plasma tube was centrifuged for 15 min at 2000*g* at 4 °C. Both plasma and whole blood samples were stored at −20 °C until further analysis.

### 2.4 Materials

Solanidine (13 264‐1MG; Sigma), formic acid (84865.180, HiPerSolv; VWR Chemicals), methanol (ultra‐gradient HPLC grade, 8402; J.T. Baker), water (LiChrosolv, liquid chromatography‐mass spectrometry [LC‐MS] grade, 1.15333.2500; Merck) and dextrometorphan‐d3 solution, 100 μg/mL (D‐071‐1ML; Sigma Aldrich) were used for LCMS‐analysis. We used the following reagents for CYP2D6 genotyping (all Thermo Fischer Scientific): *2 (rs16947; C__27102425_50), *3 (rs35742686; C__32407232_L0), *4 (rs3892097; C__27102431_D0), *6 (rs5030655; C__32407243_20), *8 (rs5030865; C_30634117C_K0), *9 (rs5030656; C__32407229_60), *10 (rs1065852; C__11484460_40), *14 (rs5030865; C_30634117D_M0), *17 (rs28371706; C___2222771_A0), *41 (rs28371725; C__34816116_20) and copy number variation assay (Hs00010001_cn). CYP2D6*1 supersomes (456 217; Corning) and **nicotinamide adenine dinucleotide phosphate** tetrasodium (16.156.500; Biomol) were used for the CYP2D6 biocatalysis.

Charcoal stripped human plasma was prepared as follows.^40^ First, 45 mL of plasma from three unrelated healthy donors (two female, one male) was vortexed and 0.45 g of activated charcoal was added. The suspension was vortexed for 5 min and rotated at 10 rotations per minute for 16 h at 4 °C. The solution was centrifuged three times at 4000*g* for 1 h at 4 °C and the supernatant collected.

4‐OH‐solanidine was synthesized with solanidine as the starting substance, analogous to the synthesis of 4‐OH‐cholesterol beginning from **cholesterol** , as described previously.^41^, ^42^ In short, solanidine was oxidized by selenium dioxide in dioxane in the presence of formic acid to 4‐OH‐solanidine and isolated by flash chromatography on silica gel (**Supporting Information Data** **S2**). After purification the purity was >95% based on high‐performance liquid chromatography‐charged aerosol detector (HPLC‐CAD) peak areas.

### 2.5 LC/MS analysis of solanidine and its metabolites

Liquid chromatography/mass spectrometry (LC/MS) analysis was performed using an Agilent 1290 Infinity II UHPLC coupled to a SCIEX QTRAP6500+ triple quadrupole mass spectrometer. The LC/MS method used for quantification of solanidine and its metabolites was analogous to a method described previously,^29^ with minor modifications. Modifications included the use of a standard for measurements of the metabolite 4‐OH‐solanidine, while SSDA measurements depended on the measured area of the chromatographic peaks, as described earlier.^26^ In brief, sample preparation was as follows: 25 μL of plasma was protein precipitated with 100 μL of 0.1% (v/v) formic acid and 1 ng mL^−1^ dextromethorphan‐d_3_ (load control) in methanol and vortexed for 10 s. The sample was left in a refrigerator at −20 °C for 1 h. The sample was centrifuged for 30 min at 17000*g* at 4 °C, then 65 μL of supernatant was transferred to an HPLC vial containing an insert. The injection volume was 12 μL.

The following multiple reaction monitoring (MRM) transitions were used for quantification: solanidine *m/z* 398.3 ➔ 98.1, 4‐OH‐solanidine *m/z* 414.3 ➔ 98.1, SSDA *m/z* 444.3 ➔ 370.3. The following MRM transitions were used for identification: dextromethorphan‐d_3_ *m/z* 275.2 ➔ 215.2, solanidine *m/z* 398.3 ➔382.3, 4‐OH‐solanidine *m/z* 414.3 ➔ 398.3, SSDA *m/z* 444.3 ➔98.1.

Solanidine and 4‐OH‐solanidine were quantified in a linear range of 0.015‐30 ng mL^−1^ in charcoal stripped human plasma as the matrix with a weighting of 1/*x* ^2^ and resulting in *r* ^2^ = 0.992 for solanidine and *r* ^2^ = 0.989 for 4‐OH‐solanidine.

The 4‐OH‐solanidine levels of the standards were based on dried, weighed substance. No correction for lower purity was undertaken.

### 2.6 Solanidine biocatalysis

First, 10 ng mL^−1^ solanidine was incubated in 1 mM nicotinamide adenine dinucleotide phosphate tetrasodium, 2.5 pM CYP2D6*1, and 25 mM potassium phosphate buffer at pH 7.4. Incubation was at 37 °C and was performed in triplicate. Workup and LC‐MS analysis were analogous to plasma samples.

### 2.7 Genotyping

CYP2D6 genotyping was performed using a QuantStudio 6 Pro qPCR‐Machine (Applied Biosystems) and TaqMan assays according to the manufacturer's instructions. The following CYP2D6 variants were assessed: *2 (rs16947), *3 (rs3574268), *4 (rs3892097), *6 (rs5030655), *8 (rs5030865), *9 (rs5030656), *10 (rs1065852), *14 (rs5030865), *17 (rs28371706) and *41 (rs28371725). Copy number variation, ie, duplication (*xN) and deletion (*5), was assessed using a FAM™ dye‐labelled minor groove binder probe and unlabelled PCR primers. CYP2D6 activity scores (AS) were calculated for each patient based on the current recommendation of the Clinical Pharmacogenetics Implementation Consortium. Metabolic phenotypes (genotype‐predicted phenotypes) were assigned to patients based on their diplotypes.^43^, ^44^

### 2.8 Calculating the shift in AS

We aimed to quantify the genotype‐phenotype mismatch by calculating individual shifts from the AS by comparing the measurements of CYP2D6 biomarkers in plasma with the genotyping results. We excluded poor metabolizers (PMs) from the analysis because enzyme inhibition in the absence of functional CYP2D6 alleles is biologically implausible because the PM can be characterized by a gene deletion, meaning no enzyme function and thus no metabolites that can be formed.

The shift in AS was calculated as follows. First the natural logarithm of the metabolic ratio (ln MR) SSDA/solanidine was obtained.^29^ To enable calculation of ln MR SSDA/solanidine in patients where no metabolite or solanidine could be detected, we imputed the lowest area measured divided by two and when calculating the ln MR 4‐OH‐solanidine/solanidine in patients where no metabolite or solanidine could be detected, we imputed the lower limit of quantification divided by two.^26^, ^29^ The equation of the linear regression of the ln MR against the AS, including the slope *m* and the offset *t*, was then retrieved.

lnMR=m·AS+t

Manipulation of the formula allowed a new activity score based on phenotyping (AS*) to be retrieved:

$$\mathrm{AS}*=\left(\ln\mathrm{MR}-t\right)\cdot m^{-1}$$

Contrasting these two AS values allowed the quantification of the phenoconversion. Hence, the shift in AS is defined as follows:

Shift inAS=AS*–AS

therefore:

$$\text{Shift in}\;\mathrm{AS}=\left(\ln\;\mathrm{MR}-t\right)\cdot m^{-1}-\mathrm{AS}$$

We repeated the calculation using the ln MR 4‐OH‐solanidine/solanidine, thus calculating two shifts in AS, one depending on measurement of ln MR SSDA/solanidine and one on measurement of ln MR 4‐OH‐solanidine/solanidine.

There is a known variation in CYP2D6 activity within the same genotypes.^45^ We therefore attempted to explain parts of the CYP2D6 variability using the shift in AS as the outcome.

### 2.9 Statistical analysis

For population characteristics, absolute numbers and percentages were calculated for categorical variables. Continuous variables were checked for normal distribution by plotting histograms and using the Shapiro‐Wilk test. As continuous variables for patient characteristics were not normally distributed (*P* < .001), medians and interquartile ranges (IQR) were calculated. The Hardy‐Weinberg equilibrium (HWE) was assessed for each single nucleotide polymorphism analysed by the chi‐squared test.

When comparing groups of patients with any use of CYP2D6 substrates and inhibitors *vs* those without, the non‐parametric Mann‐Whitney *U*‐test was used to detect statistical significance. In addition, we ran one‐way ANOVA with a post hoc one‐sided Dunnett's test, assuming patients without any use of substrates or inhibitors would have a higher shift in AS to compare the shift in AS based on SSDA/solanidine and 4‐OH‐solanidine/solanidine metabolic ratios between the group without use of substrates and inhibitors (None) with the following three groups: with use of substrates and without inhibitors (Substrate), without use of substrates and with use of inhibitors (Inhibitor), and with the use of substrates and inhibitors (Substrate and inhibitor). Linear regression was used to assess the correlations of the AS with ln MR SSDA/solanidine and with ln MR 4‐OH‐solanidine/solanidine for the whole population, as well as for the population excluding PMs as predicted by the genotype. Within a secondary analysis, we aimed to weigh CYP2D6 substrates and inhibitors differently. We decided against the use of *K*_i_ or *K*_m_ values because there is a large variability in reported data,^46^ and other relevant variables for inhibition such as volume of distribution, half‐life, formation of inhibiting metabolites, plasma protein binding and mode of inhibition are sparse for patients resembling our multimorbid, geriatric cohort. We calculated a weighted inhibition score for the use in the above‐described models instead of the number of CYP2D6 substrates and inhibitors. To this end, we counted weak inhibitors as 1, moderate inhibitors as 2, according to expected area under the curve values depicted by the Flockhart table,^37^ and substrates as 0.5 to describe their lower inhibitory potential, as done in previous analyses.^13^, ^24^ *P* values were analysed to detect significant correlations.

Multiple regression was used to assess the association of the use of CYP2D6 substrates and inhibitors with the calculated shifts in AS adjusting for potential confounders in a geriatric population. To this end, we used a hypothesis‐driven approach. We calculated three models, adjusting stepwise for potential confounders. First, a crude beta‐estimate for the use of CYP2D6 substrates/inhibitors was calculated. Second, the model was adjusted for age and sex to adjust for typically relevant demographic variables. Third, the model was adjusted for eGFR and CCI. While using metabolic ratios for hepatic elimination, we aimed to likewise adjust for differences in renal elimination. Thus, we used eGFR as variable. To account for patient comorbidity, we also included the CCI in the model.^46^ Beta‐estimates with corresponding 95% confidence intervals (CIs) were calculated. All statistical analyses were conducted using GraphPad Prism 10.2.3. An alpha level below 0.05 was deemed statistically significant.

### 2.10 Nomenclature of targets and ligands

Key protein targets and ligands in this article are hyperlinked to corresponding entries in http://www.guidetopharmacology.org, the common portal for data from the IUPHAR/BPS Guide to PHARMACOLOGY, and are permanently archived in the Concise Guide to PHARMACOLOGY 2019/20.^47^

## 3 RESULTS

**FIGURE 1:**
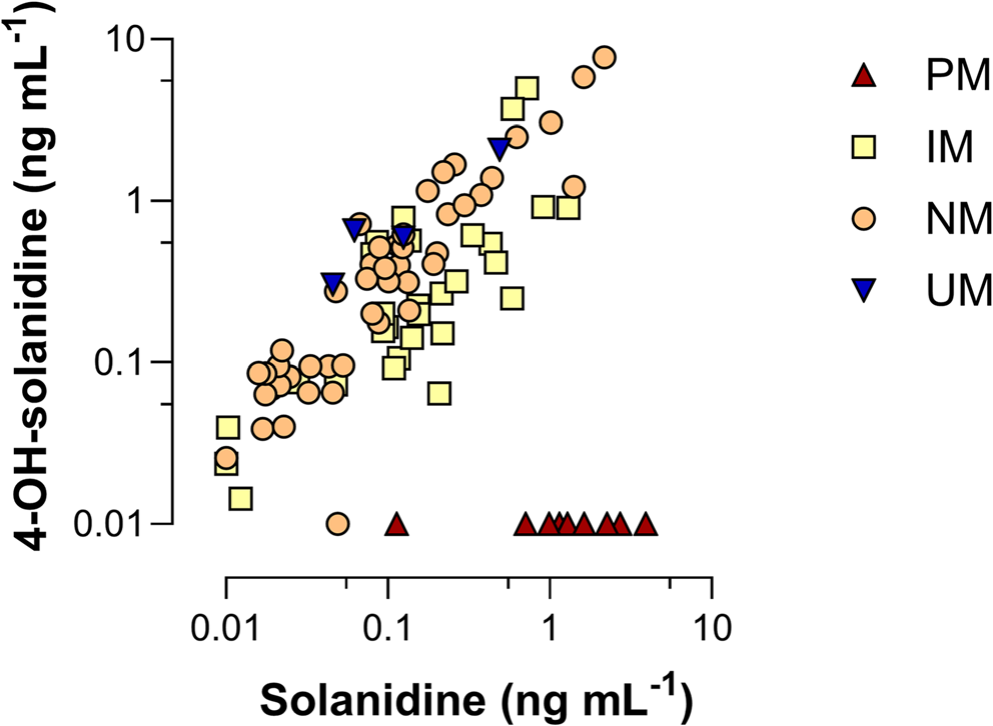
Solanidine and 4‐OH‐solanidine plasma levels as measured via liquid chromatography tandem mass spectroscopy in the cohort (N = 88). Genotype‐predicted phenotypes are shown in colour. Undetected levels are printed as 0.01 ng mL^‐1^. PM, poor metabolizer; IM, intermediate metabolizer; NM, normal metabolizer; UM, ultra rapid metabolizer.

A total of 90 patients were analysed. Solanidine or its metabolites SSDA and 4‐OH‐solanidine could be quantified in 88 out of 90 patient samples. Thus, 88 patients were included in the primary analysis. The synthesized 4‐OH‐solanidine standard had the same retention time for the characteristic transitions as plasma samples and solanidine incubated with CYP2D6 supersomes (**Supporting Information Data** **S3**). Patient characteristics are shown in Table 1. Solanidine and 4‐OH‐solanidine were quantified in the cohort using external calibration with standards, and the plasma levels are shown in Figure 1, while SSDA was quantified using the chromatographic peak areas and is shown in **Supporting Information Data** **S4**. The median age of the cohort was 83 years (IQR 77‐87) and the majority, 70.5% (n = 62) were female. Of the single nucleotide polymorphisms assessed, nine out of 10 were in the HWE. One marker, namely *10 (rs1065852), showed statistically significant deviation (*P* = .023) from the HWE in our cohort. A list of the corresponding diplotypes together with their respective frequency, predicted phenotypes and assigned AS can be found in **Supporting Information Data** **S5**.

**TABLE 1:** Descriptive characteristics of the study population of a geriatric, multimedicated cohort (N = 88).

	Missing values, n (%)	Characteristics
Age (years), median (IQR)	‐	83 (77‐87)
Female sex, n (%)	‐	62 (70.5)
Caucasian descent, n (%)	‐	87 (98.9)
No. of medications, median (IQR)	‐	15 (12‐17)
No. of CYP2D6 substrates/inhibitors, median (IQR)	‐	1 (0‐2)
No. of CYP2D6 substrates, n (%)	‐	
0		40 (45.5)
1		36 (40.9)
≥2		12 (13.6)
No. of CYP2D6 inhibitors, n (%)	‐	
0		64 (72.7)
1		23 (26.1)
≥2		1 (1.1)
Solanidine levels (ng mL^−1^), median (IQR)	‐	0.12 (0.05‐0.42)
4‐OH‐solanidine levels (ng mL^−1^), median (IQR)	‐	0.24 (0.07‐0.61)
Parker mobility score, median (IQR)	7 (8.0)	4 (2‐6)
Geriatric Depression Scale, median (IQR)	32 (36.4)	3 (0‐6)
Hand strength (Newton), median (IQR)	23 (26.1)	16.3 (12.6‐22)
Fibrosis‐4 Index, median (IQR)	5 (5.6)	1.63 (1.19‐2.29)
Estimated glomerular filtration rate (mL min^−1^ 1.73 m^−2^), median (IQR)	‐	57.2 (44.8‐73.0)
Genotype‐predicted phenotypes, n (%)	‐	
Poor metabolizers		9 (10.2)
Intermediate metabolizers		29 (33.0)
Normal metabolizers		46 (52.3)
Ultra‐rapid metabolizers		4 (4.5)
Charlson Comorbidity Index, median (IQR)	‐	6 (5‐8)
Disease, n (%)	‐	
Type 2 diabetes		39 (44.3)
Myocardial infarction		40 (45.5)
Heart failure		21 (23.9)
Peripheral artery disease		12 (13.6)
Stroke or transient ischemic attack		19 (21.6)
Dementia		9 (10.2)
Chronic obstructive pulmonary disease		24 (27.3)
Gastritis		24 (27.3)
Liver disease		8 (9.1)
Hemiplegia or hemiparesis		3 (3.4)
Solid tumour or leukaemia or lymphoma		24 (27.3)

Abbreviations: IQR, interquartile range; CYP2D6, cytochrome P450 2D6.

**FIGURE 2:**
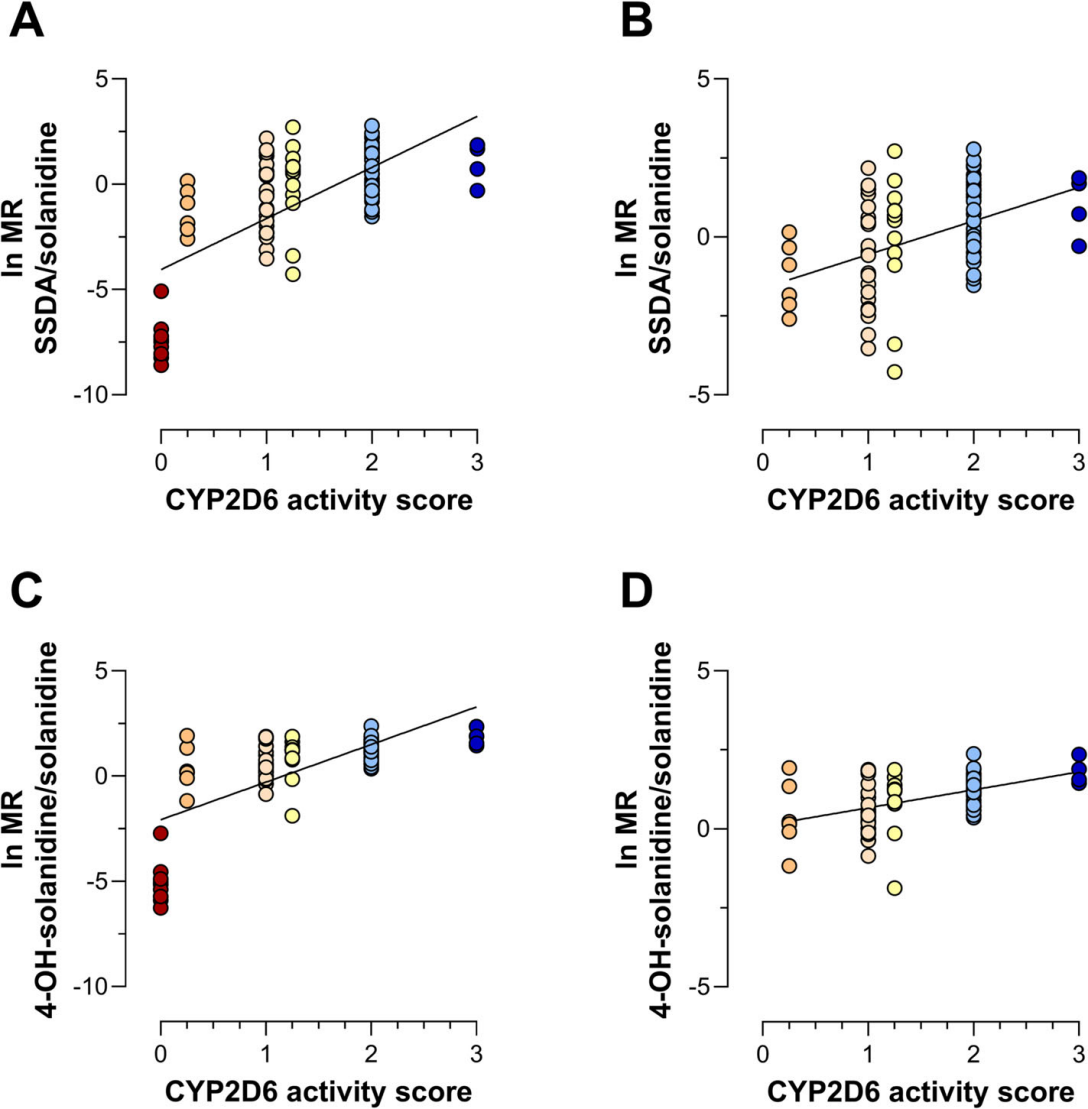
Linear regression of the natural logarithm of the metabolic ratio (ln MR) versus genotype‐predicted CYP2D6 activity scores in a population of geriatric, multimedicated patients using the metabolic ratio: (A) 3,4‐seco‐solanidine‐3,4‐dioic acid (SSDA)/solanidine and including CYP2D6 poor metabolizers (N = 88, *P* < .001, *R* ^2^ = 0.464), (B) 3,4‐seco‐solanidine‐3,4‐dioic acid (SSDA)/solanidine and excluding CYP2D6 poor metabolizers (N = 79, *P* < .001, *R* ^2^ = 0.183), (C) 4‐OH‐solanidine/solanidine and including poor metabolizers (N = 88, *P* < .001, *R* ^2^ = 0.469) and (D) 4‐OH‐solanidine/solanidine and excluding poor metabolizers (N = 79, *P* < .001, *R* ^2^ = 0.217). Coloring is based on CYP2D6 activity scores. CYP2D6, cytochrome P450 2D6; ln, natural logarithm; MR, metabolic ratio; SSDA, 3,4‐seco‐solanidine‐3,4‐dioic acid.

The metabolite SSDA could not be detected in any PM and in one non‐PM, which showed a lower solanidine level than all the PMs in our dataset. The ln MR SSDA/solanidine and ln MR 4‐OH‐solanidine/solanidine correlated significantly with the AS, both with (*P* < .001) and without (*P* < .001) inclusion of PMs. While PMs were clearly separated, other phenotype groups overlapped when analysing solanidine MRs (Figure 2).

**FIGURE 3:**
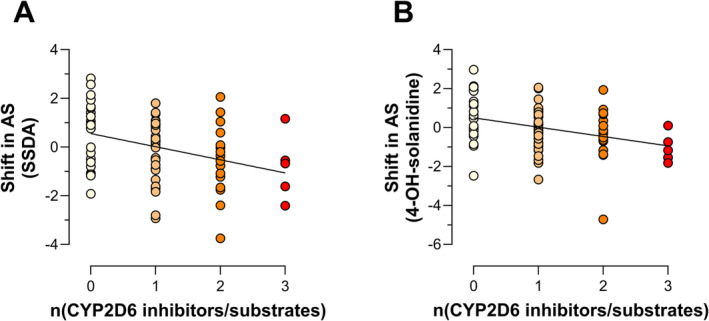
Linear regression of the shift in activity score versus the numbers of CYP2D6 substrates and inhibitors documented in the patients’ medication with the shift in AS calculated based on metabolic ratios of a) SSDA/solanidine (*P* = .002, *R* ^2^ = 0.119) and (B) 4‐OH‐solanidine/solanidine (*P* = 0.002, *R* ^2^ = 0.117). Coloring is based on number of CYP2D6 inhibitors/substrates. AS, activity score; CYP2D6, cytochrome P450 2D6; SSDA, 3,4‐seco‐solanidine‐3,4‐dioic acid.

There was a high amount of drug use observed in the cohort, with a median of 15 drugs (IQR 12‐17), along with high morbidity, as depicted by a median CCI of 6 (IQR 5‐8). Patients in the cohort took a median of one (IQR 0‐2) drug classified as a CYP2D6 substrate or inhibitor. The use of CYP2D6 substrates/inhibitors in the cohort is shown in Table 2. There was a significant difference in ln MR ratios for both SSDA/solanidine (*P* = .019) and 4‐OH‐solanidine/solanidine (*P* = .012) in patients with the use of CYP2D6 substrates and inhibitors *vs* without (**Supporting Information Data** **S6**). One‐way ANOVA showed a significant difference for the measured shift in AS based on SSDA/solanidine between the four groups (*F* = 2.874, *P* = .042), with a significant difference for no use of substrates and inhibitors *vs* use of substrates (*P* = .023) and use of inhibitors (*P* = .032) shown by Dunnett's test for multiple comparisons (**Supporting Information Data** **S7**). The use of CYP2D6 substrates and inhibitors correlated significantly with the shift in AS in univariate analysis based both on SSDA/solanidine (*P* = .002, *R* ^2^ = 0.119) (Figure 3A and **Supporting Information Data** **S8**) as well as on 4‐OH‐solanidine/solanidine measurements (Figure 3B and **Supporting Information Data** **S8**) (*P* = .002, *R* ^2^ = 0.117). When using the weighted inhibition score, we again found correlations with the shift in AS based on SSDA/solanidine (*P* = .033, *R* ^2^ = 0.057) and on 4‐OH‐solanidine/solanidine (*P* = .026, *R* ^2^ = 0.063) (**Supporting Information Data** **S9**).

**TABLE 2:** Use of cytochrome P450 (CYP) 2D6 substrates and inhibitors by the study population of geriatric, multimedicated patients (N = 88) and the weighting of the drugs used for the weighted linear regression.

Drug	Patients, n (%)	Flockhart classification	Weighting
**Amitriptyline**	2 (2.3)	Substrate	0.5
**Flecainide**	1(1.1)	Substrate	0.5
**Metoprolol**	24 (27.3)	Substrate	0.5
**Nebivolol**	3 (3.4)	Substrate	0.5
**Ondansetron**	6 (6.8)	Substrate	0.5
**Oxycodone**	18 (20.5)	Substrate	0.5
**Propranolol**	1 (1.1)	Substrate	0.5
**Tamoxifen**	1(1.1)	Substrate	0.5
**Timolol**	1(1.1)	Substrate	0.5
**Venlafaxine**	3 (3.4)	Substrate	0.5
**Metoclopramide**	1 (1.1)	in vitro evidence only	1
**Promethazine**	2 (2.3)	Unclear inhibitor	1
**Amiodarone**	8 (9.1)	Weak inhibitor	1
**Celecoxib**	1 (1.1)	Weak inhibitor	1
**Citalopram**	7 (8.0)	Weak inhibitor	1
Dimenhydrinate^a^	2 (2.2)	Weak inhibitor	1
**Escitalopram**	2(2.2)	Weak inhibitor	1
**Sertraline**	2 (2.2)	Weak inhibitor	1
**Duloxetine**	3 (3.4)	Moderate inhibitor	2

^a^ Treated like **diphenhydramine** .

The intake of CYP2D6 substrates and inhibitors was associated with a shift in AS based on SSDA measurements with a crude beta‐estimate of −0.54 (95% CI −0.87 to −0.21) (Table 3). With inclusion of age and sex into the model, the beta‐estimate was −0.54 (95% CI −0.87 to −0.21) (Model 2). After adjusting for eGFR and CCI, the beta‐estimate for the use of CYP2D6 substrates/inhibitors was −0.53 (95% CI −0.85 to −0.21) (Model 3). Next, we observed in the fully adjusted model (Model 3), smaller associations of the eGFR of −0.01 (95% CI −0.03 to 0.00) and CCI of −0.18 (95% CI −0.33 to −0.02) while age and sex were not significantly associated with the shift in AS in any model.

**TABLE 3:** Multiple linear regression models for the association of the use of CYP2D6 substrates and inhibitors with a shift in the genotype‐predicted activity score in a population of geriatric, multimedicated patients based on 3,4‐seco‐solanidine‐3,4‐dioic acid (N = 79).

	Model 1, beta‐estimate (95% CI), *R* ^2^ = 0.119 *P* = .002	Model 2, beta‐estimate (95% CI), *R* ^2^ = 0.160 *P* = .004	Model 3, beta‐estimate (95% CI), *R* ^2^ = 0.242 *P* = .001
CYP2D6 substrates/inhibitors	**−0.54 (−0.87 to −0.21)**	**−0.54 (−0.87 to −0.21)**	**−0.53 (−0.85 to −0.21)**
Age (years)	N/A	0.04 (−0.01 to 0.09)	0.04 (−0.00 to 0.09)
Female sex	N/A	−0.37 (−1.02 to 0.28)	−0,55 (−1.19 to 0.09)
eGFR (mL min^−1^ 1.73 m^−2^)	N/A	N/A	−0.01 (−0.03 to 0.00)
CCI	N/A	N/A	**−0.18 (−0.33 to −0.02)**

*Note*: Significant findings are shown in bold text.

Abbreviations: CCI, Charlson Comorbidity Index; CI, confidence interval; CYP2D6, cytochrome P450 2D6; eGFR, estimated glomerular filtration rate; N/A, not applicable.

The intake of CYP2D6 substrates and inhibitors was associated with a shift in AS based on 4‐OH‐solanidine measurements with a crude beta‐estimate of −0.48 (95% CI −0.78 to −0.18) (Table 4). With inclusion of age and sex in the model, the beta‐estimate remained −0.48 (95% CI −0.78 to −0.18) (Model 2). After adjusting for eGFR and CCI, the beta‐estimate for the use of CYP2D6 substrates/inhibitors was −0.48 (95% CI −0.78 to −0.14) (Model 3). No significant associations of age, sex, eGFR and CCI were observed.

**TABLE 4:** Multiple linear regression models for the association of the use of CYP2D6 substrates and inhibitors with a shift in the genotype‐predicted activity score in a population of geriatric, multimedicated patients based on 4‐OH‐solanidine (N = 79).

	Model 1, beta‐estimate (95% CI), *R* ^2^ = 0.117 *P* = 0.002	Model 2, beta‐estimate (95% CI), *R* ^2^ = 0.117 *P* = 0.025	Model 3, beta‐estimate (95% CI), *R* ^2^ = 0.134 *P* = 0.058
CYP2D6 substrates/inhibitors	**−0.48 (−0.78 to −0.18)**	**−0.48 (−0.78 to −0.18)**	**−0.48 (−0.78 to −0.14)**
Age (years)	N/A	0.00 (−0.04 to 0.05)	0.00 (−0.04 to 0.05)
Female sex	N/A	−0.02 (−0.62 to 0.58)	−0,09 (−0.71 to 0.52)
eGFR (mL min^−1^ 1.73 m^−2^)	N/A	N/A	−0.00 (−0.02 to 0.01)
CCI	N/A	N/A	−0.08 (−0.23 to 0.07)

*Note*: Significant findings are shown in bold text.

Abbreviations: CCI, Charlson Comorbidity Index; CI, confidence interval; CYP2D6, cytochrome P450 2D6; eGFR, estimated glomerular filtration rate; N/A, not applicable.

## 4 DISCUSSION

In this study we showed a relevant decrease of genotype‐predicted CYP2D6 activity with increasing use of CYP2D6 substrates and inhibitors. We analysed this in a multimedicated geriatric cohort with diverse pathology and a broad array of CYP2D6 interfering medications.

Our study is in line with other studies showing a strong correlation between the CYP2D6 AS and diet‐derived CYP2D6 biomarkers.^29^, ^30^ This is promising because in our study we analysed multimedicated geriatric patients instead of adults with little drug use. We were able to distinguish CYP2D6 PMs as depicted by the genotype, with both single MR SSDA/solanidine and MR 4‐OH‐solanidine/solanidine measurements. Notably, other genotype‐predicted phenotypes could not be differentiated clearly but only showed a statistical association on a population level in line with other studies calculating metabolic ratios based on **dextromethorphan** ,^26^, ^48^ tamoxifen^30^, ^49^ or solanidine^26^, ^30^, ^50^ measurements. While the turnaround time of pharmacogenetic tests is a relevant barrier to bedside implementation of pharmacogenetics and personalized medicine,^51^ such a single measurement of MR SSDA/solanidine or 4‐OH‐solanidine/solanidine might be a cost‐effective and promptly available method to detect CYP2D6 PMs in clinical routine.

On calculating the shift in the AS, we could show an additive decrease in the AS of 0.5 for each additional substrate or inhibitor used after adjusting for age, sex, renal function and multimorbidity based on models using two different solanidine metabolites. While we calculated models to adjust for potential confounders, the predictive validity of these estimates needs to be taken with caution. However, this finding is in line with Medwid et al, who also found an impact of inhibitors on the metabolic ratios SSDA/solanidine and 4‐OH‐solanidine/solanidine.^30^ We observed hints of an association of the eGFR on the shift in AS based on SSDA measurements, which might be explained by the putative positive association between kidney function and renal elimination of the probable terminal metabolite SSDA,^25^ independent of hepatic CYP2D6 activity. The CCI also showed a negative association with the shift in AS based on SSDA measurements, but as only potential confounders were added to the models, no robust interpretations can be drawn from that. Further studies with more power are needed to elucidate the impact of the different dimensions of morbidity on measured CYP2D6 activity.

A previous study by Opdam et al analysing CYP2D6 activity in healthy and frail patients found no impact of frailty on CYP2D6 activity measured by ^13^C‐dextromethorphan breath tests.^52^ We did not measure frailty in our patients, but the CCI might serve as a proxy showing high multimorbidity, which in fact suggests a high number of frail patients in our cohort. While the study of Opdam et al had difficulties achieving a sufficient sample size, we measured CYP2D6 activity in a much larger cohort. Hence, measuring solanidine‐derived biomarkers seems to be a promising tool to measure CYP2D6 activity potentially at bedside, even in frail older patients. We were able to measure CYP2D6 activity in a real‐world cohort of multimedicated and multimorbid geriatric patients, which is a clear strength of our study.

Older, multimedicated patients are at high risk for ADRs.^53^, ^54^ While the prevalence of ADRs can be reduced using pharmacogenetic diagnostics and subsequent treatment modifications, as shown in the PREPARE study,^15^ it is important to detect phenoconversion to identify situations in which a treatment recommendation might not be advantageous to a patient. With this study we could show that it is possible to detect phenoconversion by measurement of simple single MR SSDA/solanidine and/or 4‐OH‐solanidine/solanidine. CYP2D6 is involved in the occurrence of several ADRs, and its timely activity measurement is an unmet medical need.^55^ Our study shows promising results as methods to optimize drug treatment in multimedicated, geriatric patients are urgently needed.

This study has several limitations. We did not assess rare variants of the CYP2D6 pharmacogene and the nuclear factor 1B (NFIB) rs28379954 T>C polymorphism, which has recently been shown to affect nutrimetrically measured CYP2D6 activity.^56^ However, we expect only a small deviation from the AS we measured here. In addition, we chose to classify CYP2D6 interfering drugs based on the Flockhart table^37^ because many clinically relevant drugs are included. However, some drugs taken by the patients, such as melperone, were not classified as inhibitors despite strong evidence from other studies,^57^, ^58^, ^59^ which may cause an underestimation of the effect of inhibitors. Likewise, there is a high number of in vitro studies depicting *K*_i_ and *K*_m_ values with only moderate reliability between studies.^46^ Thus, we decided to not use data from in vitro studies, but focus on available in vivo data. However, this method again has limitations.^60^ We were not able to conduct subgroup analyses discriminating weak, moderate and strong CYP2D6 inhibitors due to low sample size. However, compared to a previous study with frail older adults,^52^ we were able to enrol a much higher number of patients in our cohort study. Even though we did adjust for age, sex, renal function and multimorbidity, we cannot exclude residual confounding of the results and must be cautious to draw causal conclusions. However, the fully adjusted model based on SSDA measurements, which we calculated to adjust for potential confounders, showed *R* ^2^ of 24.2% in the multiple linear regression model, which corresponds to a moderate effect according to Cohen.^61^

In conclusion, we showed the feasibility of minimally invasive CYP2D6 phenotyping in geriatric, multimedicated patients and a relevant phenoconversion with the use of CYP2D6 substrates and inhibitors. These results might serve as a prerequisite for the validation and establishment of a bedside method to measure CYP2D6 activity in multimorbid patients and successful applications of personalized medicine.

## AUTHOR CONTRIBUTIONS

J.A.S., J.P.M., J.T., B.K., T.L., F.K., J.C.S. and K.S.J. conducted the research. J.A.S. wrote the first draft of the manuscript. C.B., A.R., B.K., J.C.S. and K.S.J. designed the research. J.A.S. and K.S.J. conducted the statistical analysis of the data.

## CONFLICT OF INTEREST STATEMENT

J.A.S. is a former employee of AbbVie Germany. B.K. has served as a speaker for SphingoTec/4TEEN4 Pharmaceuticals and received travel support from 4TEEN4 Pharmaceuticals and Novo Nordisk. F.K. has served as a speaker for Novo Nordisk, Lilly, AstraZeneca, DGK‐Akademie, consulted Novo Nordisk, Bayer, PricewaterhouseCoopers/Strategy&, and received travel support from Amgen, Novo Nordisk, Boehringer Ingelheim, Bayer, SphingoTec/4TEEN4 Pharmaceuticals and Lilly. All other authors declare no conflict of interest.

## Supporting information


**SUPPORTING INFORMATION DATA S1** Definition of parameters used to calculate the Charlson Comorbidity Index and corresponding ICD‐10 codes
**SUPPORTING INFORMATION DATA S2** Synthesis of 4‐OH‐solanidine
**SUPPORTING INFORMATION DATA S3** Chromatograms of (A) 10 ng mL^−1^ solanidine before incubation with CYP2D6*1 supersomes, (B) 10 ng mL^−1^ solanidine after 24 h of incubation with CYP2D6*1 supersomes, (C) a representative plasma sample and (D) 0.25 ng mL^−1^ 4‐OH‐solanidine standard
**SUPPORTING INFORMATION DATA S4** Solanidine and seco‐solanidine‐3,4‐dioic acid (SSDA) plasma levels as measured via liquid chromatography tandem mass spectroscopy in the cohort (N = 88). Genotype‐predicted phenotypes are shown in colour. Undetected levels are printed as the lowest detected area divided by two
**SUPPORTING INFORMATION DATA S5** Diplotypes of cytochrome P450 (CYP) 2D6 and respective genotype‐predicted phenotypes in the study population of geriatric, multimedicated patients (N = 88)
**SUPPORTING INFORMATION DATA S6** Natural logarithm of the metabolic ratios (ln MR) of (A) 3,4‐seco‐solanidine‐3,4‐dioic acid (SSDA)/solanidine (*P* = .019) and (B) 4‐OH‐solanidine/solanidine (*P* = .012) measured in a population of geriatric, multimedicated patients after exclusion of CYP2D6 poor metabolizers stratified to no *vs* any use of CYP2D6 substrates and inhibitors
**SUPPORTING INFORMATION DATA S7** Shift in AS based on (A) 3,4‐seco‐solanidine‐3,4‐dioic acid (SSDA)/solanidine (*F* = 2.874, *P* = .042) and (B) 4‐OH‐solanidine/solanidine (*F* = 2.165, *P* = .099) metabolic ratios in a population of geriatric, multimedicated patients after exclusion of CYP2D6 poor metabolizers stratified to four groups with and without use of CYP2D6 substrates and inhibitors
**SUPPORTING INFORMATION DATA S8** Linear regression of the shift in activity score *vs* the numbers of cytochrome P450 (CYP) 2D6 substrates and inhibitors documented in the patients' medication with the shift in AS calculated based on (A) seco‐solanidine‐3,4‐dioic acid (SSDA) measurements (*P* = .002, *R* ^2^ = 0.119) and (B) 4‐OH‐solanidine measurements (*P* = .002, *R* ^2^ = 0.117)
**SUPPORTING INFORMATION DATA S9** Linear regression of the shift in activity score (AS) *vs* the numbers of cytochrome P450 (CYP) 2D6 substrates and inhibitors documented in the patients' medication using a weighted inhibition score. Calculation of shift in AS based on (A) 3,4‐seco‐solanidine‐3,4‐dioic acid (SSDA) measurements (*P* = .033, *R* ^2^ = 0.057) and (B) 4‐OH‐solanidine measurements (*P* = .026, *R* ^2^ = 0.063)

## Data Availability

The data that support the findings of this study are available on reasonable request from the corresponding author.
